# Supplementary figures and images for: Efficient Broadband Light-Trapping Structures on Thin-Film Silicon Fabricated by Laser, Chemical and Hybrid Chemical/Laser Treatments
Source: Materials (Basel). 2023 Mar 15;16(6):2350. doi: 10.3390/ma16062350 (PMC10056786; doi:10.3390/ma16062350)

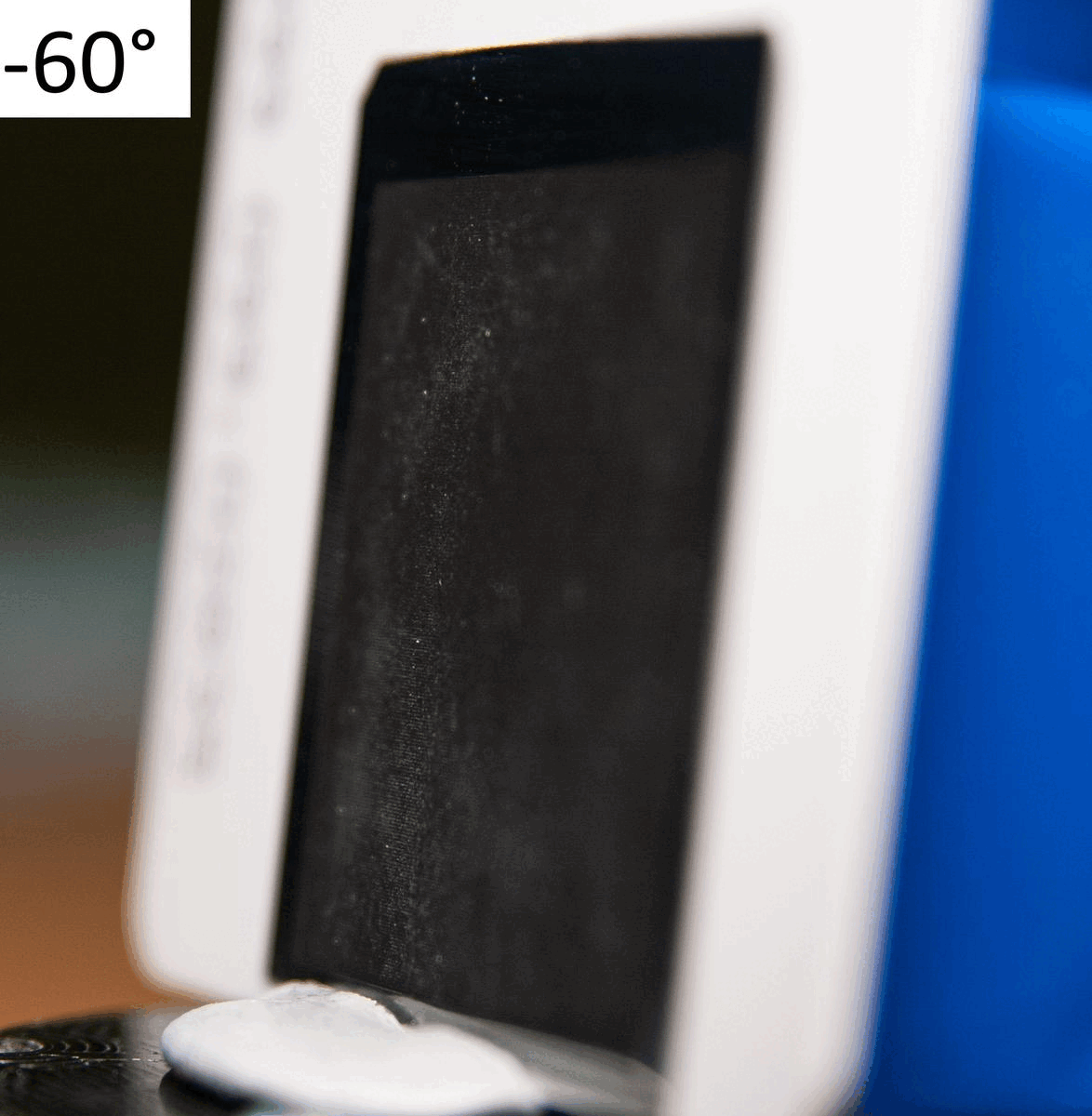

Supplement: Supplementary file 1 [file materials-16-02350-s001.zip › (Video S1) Sample #1 ¿C laser texturing of a silicon cut.gif]

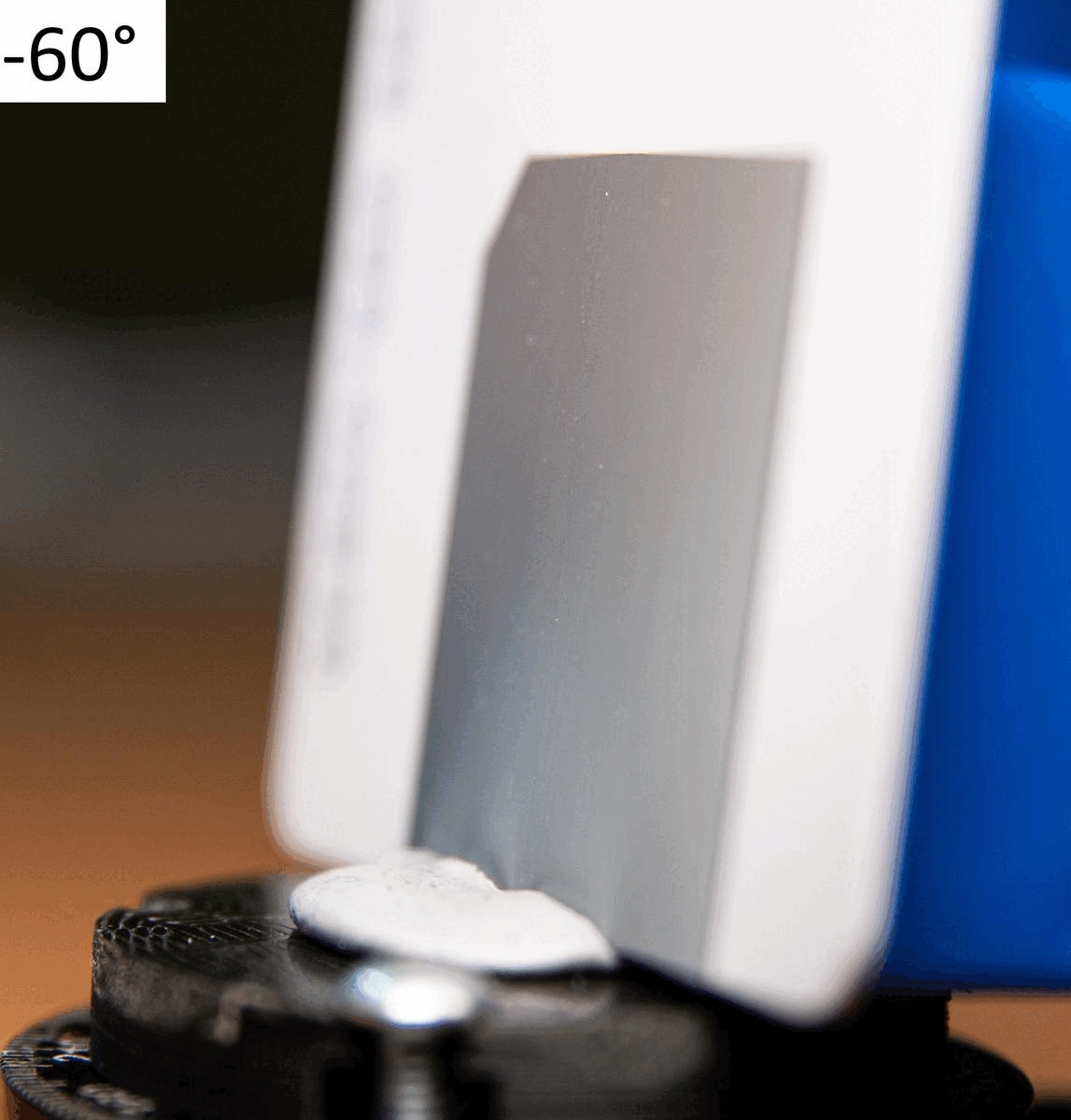

Supplement: Supplementary file 1 [file materials-16-02350-s001.zip › (Video S2) Sample #2 ¿C chemical etching.gif]

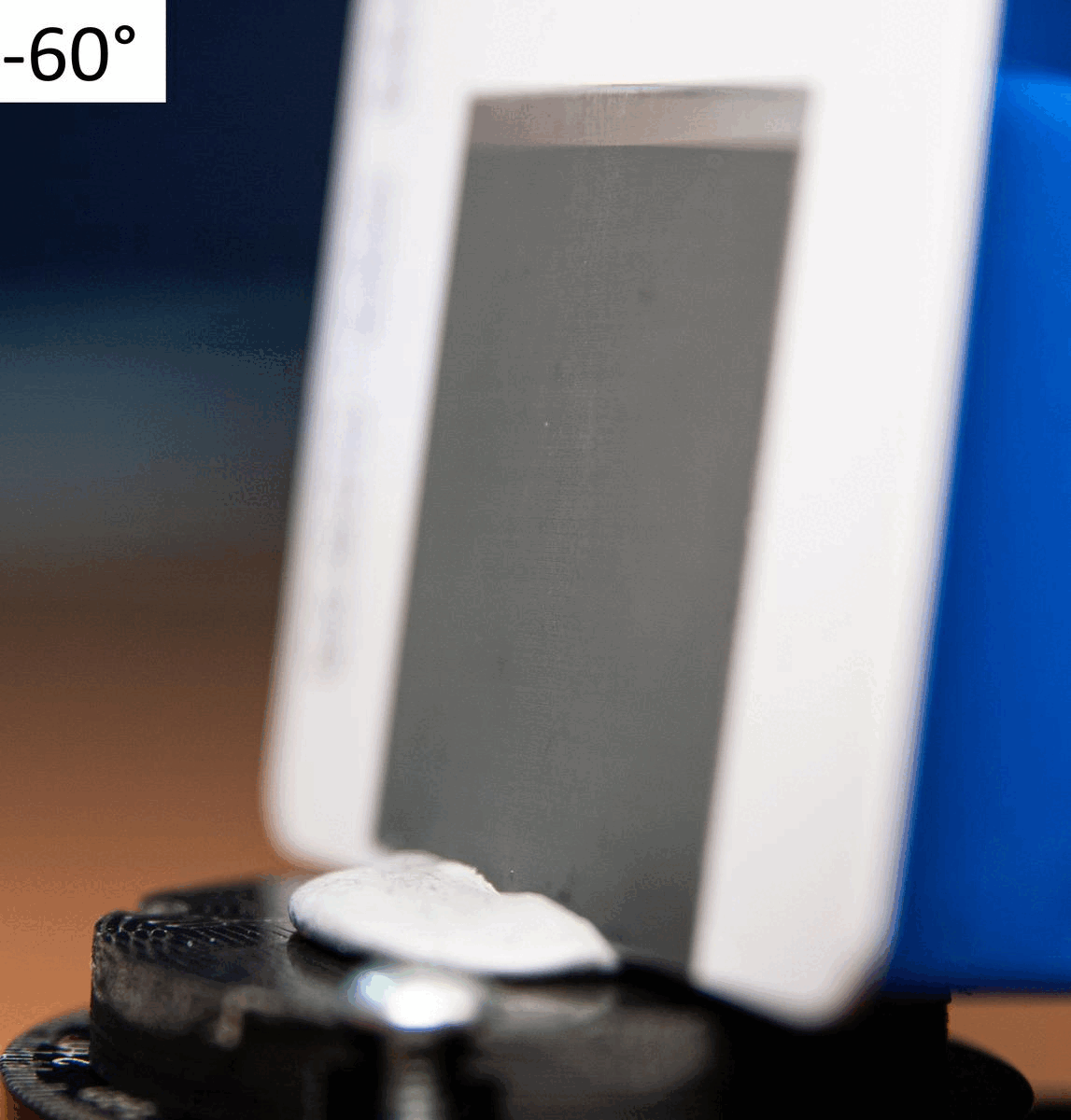

Supplement: Supplementary file 1 [file materials-16-02350-s001.zip › (Video S3) Sample #3 ¿C hybrid chemical laser fabrication.gif]
